# *N*,*N*,*N*′,*N*′-Tetra­methyl­guanidinium tetra­phenyl­borate

**DOI:** 10.1107/S160053681204860X

**Published:** 2012-11-30

**Authors:** Ioannis Tiritiris

**Affiliations:** a Fakultät Chemie/Organische Chemie, Hochschule Aalen, Beethovenstrasse 1, D-73430 Aalen, Germany

## Abstract

In the title salt, C_5_H_14_N_3_^+^·C_24_H_20_B^−^, the C—N bond lengths in the central CN_3_ unit are 1.3322 (11), 1.3385 (12) and 1.3422 (12) Å, indicating partial double-bond character. The central C atom is bonded to the three N atoms in a nearly ideal trigonal-planar geometry [N—C—N angles = 119.51 (8), 119.81 (9) and 120.69 (8)°] and the positive charge is delocalized in the CN_3_ plane. The bond lengths between the N atoms and the terminal methyl groups all have values close to a typical single bond [1.4597 (12)–1.4695 (13) Å]. The crystal packing is caused by electrostatic inter­actions between cations and anions.

## Related literature

For related structures, see: Fischer & Jones (2002 ▶); Berg *et al.* (2010 ▶); Tiritiris *et al.* (2011 ▶); Criado *et al.* (2000 ▶); Kanters *et al.* (1992 ▶); Bujak *et al.* (1999 ▶); Wong *et al.* (2004 ▶); Pajzderska *et al.* (2002 ▶).
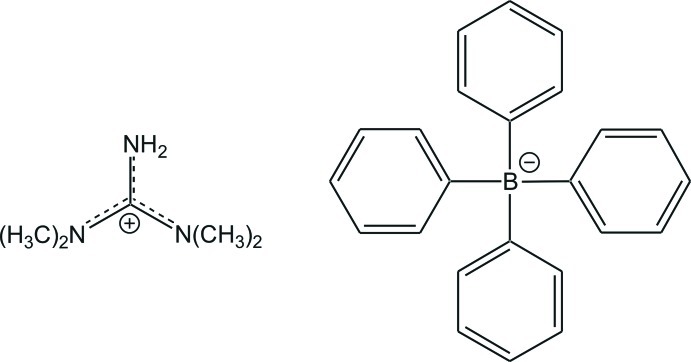


## Experimental

### 

#### Crystal data

C_5_H_14_N_3_^+^·C_24_H_20_B^−^
*M* *_r_* = 435.40
Monoclinic, 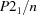

*a* = 10.9512 (5) Å
*b* = 18.1315 (9) Å
*c* = 12.5453 (7) Å
β = 96.594 (2)°
*V* = 2474.5 (2) Å^3^
*Z* = 4
Mo *K*α radiation
μ = 0.07 mm^−1^
*T* = 100 K
0.23 × 0.16 × 0.12 mm

#### Data collection

Bruker Kappa APEXII DUO diffractometer
52874 measured reflections
7573 independent reflections
6738 reflections with *I* > 2σ(*I*)
*R*_int_ = 0.021

#### Refinement

*R*[*F* ^2^ > 2σ(*F* ^2^)] = 0.041
*wR*(*F* ^2^) = 0.112
*S* = 1.04
7573 reflections
310 parameters
H atoms treated by a mixture of independent and constrained refinement
Δρ_max_ = 0.40 e Å^−3^
Δρ_min_ = −0.31 e Å^−3^

### 

Data collection: *APEX2* (Bruker, 2008 ▶); cell refinement: *SAINT* (Bruker, 2008 ▶); data reduction: *SAINT*; program(s) used to solve structure: *SHELXS97* (Sheldrick, 2008 ▶); program(s) used to refine structure: *SHELXL97* (Sheldrick, 2008 ▶); molecular graphics: *DIAMOND* (Brandenburg & Putz, 2005 ▶); software used to prepare material for publication: *SHELXL97*.

## Supplementary Material

Crystal structure: contains datablock(s) I, global. DOI: 10.1107/S160053681204860X/ff2091sup1.cif

Structure factors: contains datablock(s) I. DOI: 10.1107/S160053681204860X/ff2091Isup2.hkl

Additional supplementary materials:  crystallographic information; 3D view; checkCIF report

## Figures and Tables

**Fig. 1.**
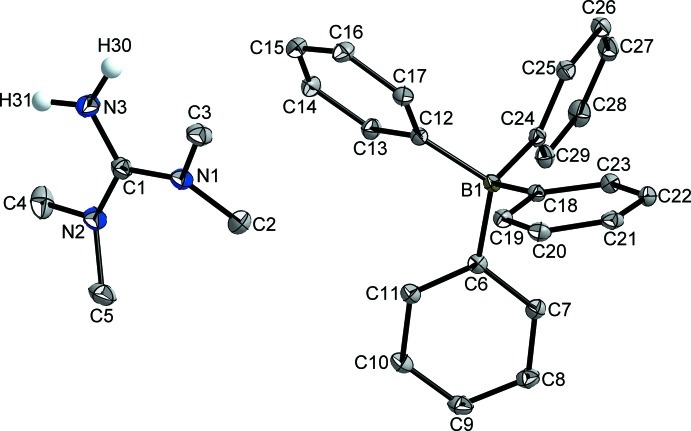
The structure of the title compound with atom labels and 50% probability displacement ellipsoids.

## supplementary crystallographic information

### Comment

Salts with the *N*,*N*,*N*',*N*'-tetramethylguanidinium ion (tmg^+^) are usually synthesized by protonation of the base *N*,*N*, *N*',*N*'-tetramethylguanidine with the appropriate acids. Until now, the crystal structures of several tmgX salts were elucidated (tmgCl: Fischer & Jones, 2002; tmgBr: Berg *et al.*, 2010; tmgHCO_3_: Tiritiris *et al.*, 2011; tmgH_2_PO_4_: Criado *et al.*, 2000; tmg^+^ pentachlorophenolate as complex with pentachlorophenol: Kanters *et al.*, 1992; tmgSbCl_4_: Bujak *et al.*, 1999). Starting from the salt tmgCl (Fischer & Jones, 2002) by reacting with sodium tetraphenylborate, it was possible to achieve an anion exchange and to obtain the title compound. According to the structure analysis, the C1–N1 bond is 1.3385 (12) Å, C1–N2 = 1.3322 (11) Å and C1–N3 = 1.3422 (12) Å, showing partial double-bond character. The N–C1–N angles are: 120.69 (8)° (N1–C1–N2), 119.51 (8)° (N1–C1–N3) and 119.81 (9)° (N2–C1–N3), which indicates a nearly ideal trigonal-planar surrounding of the carbon centre by the nitrogen atoms (Fig. 1). The positive charge is completely delocalized on the CN_3_ plane. The bonds between the N atoms and the terminal *C*-methyl groups, all have values close to a typical single bond [1.4597 (12)–1.4695 (13) Å]. The bond lengths and angles in the tetraphenylborate ion are in good agreement with the data from the crystal structure analysis of potassium tetraphenylborate (Wong *et al.*, 2004) or rubidium tetraphenylborate (Pajzderska *et al.*, 2002). Since there exist no hydrogen bonds in the title compound, crystal packing is caused by electrostatic interactions between cations and anions.

### Experimental

The title compound was obtained in an anion exchange reaction by reacting 2.0 g (13 mmol) of *N*,*N*,*N*',*N*'-tetramethylguanidinium chloride (Fischer & Jones, 2002) with 4.45 g (13 mmol) of sodium tetraphenylborate in 50 ml of acetonitrile at room temperature. After heating the mixture for 10 minutes at 353 K, the precipitated sodium chloride was filtered off. After evaporation of the solvent a colorless solid has been obtained. The title compound was recrystallized from a saturated acetonitrile solution and after several days at 273 K, colorless single crystals were formed. Yield: 5.2 g (92%).

### Refinement

The N-bound H atoms were located in a difference Fourier map and were refined freely [N—H = 0.90 (2)–0.91 (2) Å]. The hydrogen atoms of the methyl groups were allowed to rotate with a fixed angle around the C–N bond to best fit the experimental electron density, with *U*(H) set to 1.5 *U*_eq_(C) and d(C—H) = 0.98 Å. The H atoms in the aromatic rings were placed in calculated positions with (C—H) = 0.95 Å. They were included in the refinement in the riding model approximation, with *U*(H) set to 1.2 *U*_eq_(C).

### Crystal data

**Table:**

C_5_H_14_N_3_^+^·C_24_H_20_B^−^	*F*(000) = 936
*M**_r_* = 435.40	*D*_x_ = 1.169 Mg m^−^^3^
Monoclinic, *P*2_1_/*n*	Mo *K*α radiation, λ = 0.71073 Å
Hall symbol: -P 2yn	Cell parameters from 52874 reflections
*a* = 10.9512 (5) Å	θ = 2.0–30.6°
*b* = 18.1315 (9) Å	µ = 0.07 mm^−^^1^
*c* = 12.5453 (7) Å	*T* = 100 K
β = 96.594 (2)°	Block, colourless
*V* = 2474.5 (2) Å^3^	0.23 × 0.16 × 0.12 mm
*Z* = 4	

### Data collection

**Table:**

Bruker Kappa APEXII DUO diffractometer	6738 reflections with *I* > 2σ(*I*)
Radiation source: sealed tube	*R*_int_ = 0.021
Graphite monochromator	θ_max_ = 30.6°, θ_min_ = 2.0°
φ scans, and ω scans	*h* = −15→15
52874 measured reflections	*k* = −25→25
7573 independent reflections	*l* = −12→17

### Refinement

**Table:**

Refinement on *F*^2^	Primary atom site location: structure-invariant direct methods
Least-squares matrix: full	Secondary atom site location: difference Fourier map
*R*[*F*^2^ > 2σ(*F*^2^)] = 0.041	Hydrogen site location: difference Fourier map
*wR*(*F*^2^) = 0.112	H atoms treated by a mixture of independent and constrained refinement
*S* = 1.04	*w* = 1/[σ^2^(*F*_o_^2^) + (0.0559*P*)^2^ + 0.9205*P*] where *P* = (*F*_o_^2^ + 2*F*_c_^2^)/3
7573 reflections	(Δ/σ)_max_ < 0.001
310 parameters	Δρ_max_ = 0.40 e Å^−^^3^
0 restraints	Δρ_min_ = −0.31 e Å^−^^3^

### Special details

**Table:**

Geometry. All e.s.d.'s (except the e.s.d. in the dihedral angle between two l.s. planes) are estimated using the full covariance matrix. The cell e.s.d.'s are taken into account individually in the estimation of e.s.d.'s in distances, angles and torsion angles; correlations between e.s.d.'s in cell parameters are only used when they are defined by crystal symmetry. An approximate (isotropic) treatment of cell e.s.d.'s is used for estimating e.s.d.'s involving l.s. planes.
Refinement. Refinement of *F*^2^ against ALL reflections. The weighted *R*-factor *wR* and goodness of fit *S* are based on *F*^2^, conventional *R*-factors *R* are based on *F*, with *F* set to zero for negative *F*^2^. The threshold expression of *F*^2^ > σ(*F*^2^) is used only for calculating *R*-factors(gt) *etc*. and is not relevant to the choice of reflections for refinement. *R*-factors based on *F*^2^ are statistically about twice as large as those based on *F*, and *R*- factors based on ALL data will be even larger.

### Fractional atomic coordinates and isotropic or equivalent isotropic displacement parameters (Å^2^)

**Table:**

	*x*	*y*	*z*	*U*_iso_*/*U*_eq_	
N1	0.74190 (7)	0.06305 (5)	0.71562 (6)	0.01888 (15)	
N2	0.60284 (7)	0.13924 (5)	0.78860 (6)	0.02054 (16)	
N3	0.80829 (8)	0.14087 (5)	0.85684 (7)	0.02289 (17)	
H30	0.8870 (16)	0.1353 (9)	0.8435 (13)	0.041 (4)*	
H31	0.7911 (15)	0.1668 (9)	0.9157 (13)	0.038 (4)*	
C1	0.71704 (8)	0.11437 (5)	0.78672 (7)	0.01750 (16)	
C2	0.67627 (9)	0.06015 (6)	0.60739 (8)	0.02425 (19)	
H2A	0.6199	0.1022	0.5968	0.036*	
H2B	0.6294	0.0141	0.5983	0.036*	
H2C	0.7355	0.0623	0.5546	0.036*	
C3	0.85459 (8)	0.01960 (6)	0.73372 (8)	0.02234 (18)	
H3A	0.9218	0.0463	0.7056	0.034*	
H3B	0.8423	−0.0279	0.6969	0.034*	
H3C	0.8754	0.0113	0.8109	0.034*	
C4	0.57784 (10)	0.20819 (6)	0.84245 (8)	0.0281 (2)	
H4A	0.5715	0.1984	0.9185	0.042*	
H4B	0.5004	0.2292	0.8089	0.042*	
H4C	0.6449	0.2431	0.8361	0.042*	
C5	0.49544 (9)	0.09133 (6)	0.76198 (8)	0.0262 (2)	
H5A	0.5230	0.0408	0.7510	0.039*	
H5B	0.4476	0.1091	0.6962	0.039*	
H5C	0.4441	0.0921	0.8210	0.039*	
B1	0.77207 (8)	0.10576 (5)	0.22287 (7)	0.01225 (15)	
C6	0.62087 (7)	0.10464 (4)	0.20365 (6)	0.01299 (14)	
C7	0.56097 (8)	0.08674 (5)	0.10126 (7)	0.01578 (15)	
H7A	0.6098	0.0758	0.0454	0.019*	
C8	0.43355 (8)	0.08443 (5)	0.07831 (7)	0.01832 (16)	
H8A	0.3973	0.0719	0.0082	0.022*	
C9	0.35964 (8)	0.10055 (5)	0.15814 (8)	0.02010 (17)	
H9A	0.2726	0.1000	0.1430	0.024*	
C10	0.41494 (8)	0.11741 (6)	0.26041 (8)	0.02207 (18)	
H10A	0.3654	0.1281	0.3159	0.026*	
C11	0.54315 (8)	0.11869 (5)	0.28233 (7)	0.01841 (16)	
H11A	0.5787	0.1295	0.3533	0.022*	
C12	0.82722 (7)	0.13031 (4)	0.34510 (6)	0.01287 (14)	
C13	0.78885 (8)	0.19702 (5)	0.38812 (7)	0.01620 (15)	
H13A	0.7268	0.2250	0.3471	0.019*	
C14	0.83773 (8)	0.22387 (5)	0.48811 (7)	0.01935 (17)	
H14A	0.8077	0.2687	0.5144	0.023*	
C15	0.93051 (9)	0.18509 (6)	0.54945 (7)	0.02074 (18)	
H15A	0.9638	0.2028	0.6179	0.025*	
C16	0.97343 (8)	0.12013 (5)	0.50873 (7)	0.01963 (17)	
H16A	1.0381	0.0937	0.5488	0.024*	
C17	0.92193 (8)	0.09334 (5)	0.40884 (7)	0.01579 (15)	
H17A	0.9522	0.0484	0.3832	0.019*	
C18	0.81527 (7)	0.02273 (4)	0.19171 (6)	0.01238 (14)	
C19	0.79975 (7)	−0.03773 (5)	0.25931 (6)	0.01439 (15)	
H19	0.7684	−0.0289	0.3257	0.017*	
C20	0.82836 (8)	−0.10987 (5)	0.23308 (7)	0.01630 (16)	
H20A	0.8165	−0.1490	0.2812	0.020*	
C21	0.87440 (8)	−0.12473 (5)	0.13634 (7)	0.01601 (15)	
H21	0.8955	−0.1737	0.1184	0.019*	
C22	0.88900 (8)	−0.06665 (5)	0.06645 (7)	0.01521 (15)	
H22A	0.9192	−0.0759	−0.0003	0.018*	
C23	0.85949 (7)	0.00525 (5)	0.09407 (6)	0.01382 (15)	
H23A	0.8698	0.0439	0.0448	0.017*	
C24	0.82661 (8)	0.16882 (4)	0.14711 (6)	0.01348 (14)	
C25	0.95325 (8)	0.17168 (5)	0.13683 (7)	0.01668 (16)	
H25A	1.0053	0.1345	0.1707	0.020*	
C26	1.00537 (9)	0.22663 (5)	0.07917 (7)	0.02039 (17)	
H26A	1.0912	0.2262	0.0737	0.024*	
C27	0.93187 (10)	0.28230 (5)	0.02953 (7)	0.02249 (18)	
H27A	0.9668	0.3197	−0.0106	0.027*	
C28	0.80688 (9)	0.28248 (5)	0.03943 (7)	0.02106 (18)	
H28A	0.7560	0.3207	0.0071	0.025*	
C29	0.75587 (8)	0.22642 (5)	0.09701 (7)	0.01653 (16)	
H29A	0.6701	0.2274	0.1024	0.020*	

### Atomic displacement parameters (Å^2^)

**Table:**

	*U*^11^	*U*^22^	*U*^33^	*U*^12^	*U*^13^	*U*^23^
N1	0.0149 (3)	0.0227 (4)	0.0191 (3)	0.0019 (3)	0.0022 (3)	−0.0004 (3)
N2	0.0162 (3)	0.0255 (4)	0.0199 (3)	0.0044 (3)	0.0020 (3)	0.0012 (3)
N3	0.0170 (4)	0.0286 (4)	0.0225 (4)	0.0026 (3)	−0.0004 (3)	−0.0053 (3)
C1	0.0158 (4)	0.0202 (4)	0.0166 (4)	0.0012 (3)	0.0024 (3)	0.0027 (3)
C2	0.0218 (4)	0.0321 (5)	0.0184 (4)	−0.0026 (4)	0.0003 (3)	−0.0018 (3)
C3	0.0159 (4)	0.0233 (4)	0.0280 (4)	0.0023 (3)	0.0035 (3)	−0.0016 (3)
C4	0.0294 (5)	0.0328 (5)	0.0222 (4)	0.0154 (4)	0.0027 (4)	−0.0002 (4)
C5	0.0144 (4)	0.0379 (6)	0.0262 (5)	−0.0005 (4)	0.0025 (3)	0.0076 (4)
B1	0.0119 (4)	0.0130 (4)	0.0121 (4)	0.0004 (3)	0.0021 (3)	−0.0005 (3)
C6	0.0131 (3)	0.0122 (3)	0.0138 (3)	0.0006 (3)	0.0019 (3)	0.0006 (3)
C7	0.0157 (4)	0.0173 (4)	0.0143 (3)	0.0000 (3)	0.0014 (3)	−0.0001 (3)
C8	0.0166 (4)	0.0200 (4)	0.0174 (4)	−0.0012 (3)	−0.0020 (3)	0.0019 (3)
C9	0.0126 (4)	0.0237 (4)	0.0235 (4)	−0.0002 (3)	0.0001 (3)	0.0020 (3)
C10	0.0140 (4)	0.0312 (5)	0.0217 (4)	0.0004 (3)	0.0048 (3)	−0.0035 (3)
C11	0.0142 (4)	0.0250 (4)	0.0162 (4)	−0.0002 (3)	0.0027 (3)	−0.0035 (3)
C12	0.0122 (3)	0.0137 (3)	0.0131 (3)	−0.0007 (3)	0.0030 (3)	−0.0006 (3)
C13	0.0160 (4)	0.0152 (4)	0.0173 (4)	0.0001 (3)	0.0020 (3)	−0.0024 (3)
C14	0.0192 (4)	0.0196 (4)	0.0198 (4)	−0.0037 (3)	0.0047 (3)	−0.0067 (3)
C15	0.0195 (4)	0.0267 (4)	0.0159 (4)	−0.0072 (3)	0.0014 (3)	−0.0047 (3)
C16	0.0160 (4)	0.0254 (4)	0.0167 (4)	−0.0015 (3)	−0.0017 (3)	0.0006 (3)
C17	0.0143 (3)	0.0175 (4)	0.0155 (3)	0.0006 (3)	0.0014 (3)	−0.0005 (3)
C18	0.0103 (3)	0.0136 (3)	0.0131 (3)	0.0000 (3)	0.0011 (2)	−0.0009 (3)
C19	0.0138 (3)	0.0151 (4)	0.0146 (3)	−0.0008 (3)	0.0028 (3)	−0.0001 (3)
C20	0.0160 (4)	0.0135 (4)	0.0194 (4)	−0.0018 (3)	0.0022 (3)	0.0014 (3)
C21	0.0137 (3)	0.0132 (3)	0.0210 (4)	−0.0008 (3)	0.0015 (3)	−0.0031 (3)
C22	0.0139 (3)	0.0167 (4)	0.0154 (3)	−0.0006 (3)	0.0029 (3)	−0.0036 (3)
C23	0.0136 (3)	0.0143 (3)	0.0136 (3)	−0.0003 (3)	0.0018 (3)	−0.0003 (3)
C24	0.0157 (3)	0.0128 (3)	0.0122 (3)	−0.0005 (3)	0.0030 (3)	−0.0022 (3)
C25	0.0158 (4)	0.0174 (4)	0.0173 (4)	−0.0017 (3)	0.0039 (3)	−0.0026 (3)
C26	0.0208 (4)	0.0228 (4)	0.0188 (4)	−0.0074 (3)	0.0077 (3)	−0.0048 (3)
C27	0.0317 (5)	0.0199 (4)	0.0170 (4)	−0.0089 (4)	0.0076 (3)	−0.0009 (3)
C28	0.0302 (5)	0.0153 (4)	0.0180 (4)	−0.0007 (3)	0.0038 (3)	0.0022 (3)
C29	0.0196 (4)	0.0147 (4)	0.0157 (3)	0.0007 (3)	0.0038 (3)	0.0001 (3)

### Geometric parameters (Å, º)

**Table:**

N1—C1	1.3385 (12)	C11—H11A	0.9500
N1—C3	1.4597 (12)	C12—C17	1.4050 (11)
N1—C2	1.4624 (12)	C12—C13	1.4081 (11)
N2—C1	1.3322 (11)	C13—C14	1.3939 (12)
N2—C4	1.4617 (13)	C13—H13A	0.9500
N2—C5	1.4695 (13)	C14—C15	1.3927 (13)
N3—C1	1.3422 (12)	C14—H14A	0.9500
N3—H30	0.903 (17)	C15—C16	1.3872 (14)
N3—H31	0.914 (16)	C15—H15A	0.9500
C2—H2A	0.9800	C16—C17	1.4008 (12)
C2—H2B	0.9800	C16—H16A	0.9500
C2—H2C	0.9800	C17—H17A	0.9500
C3—H3A	0.9800	C18—C23	1.4042 (11)
C3—H3B	0.9800	C18—C19	1.4083 (11)
C3—H3C	0.9800	C19—C20	1.3931 (12)
C4—H4A	0.9800	C19—H19	0.9500
C4—H4B	0.9800	C20—C21	1.3930 (12)
C4—H4C	0.9800	C20—H20A	0.9500
C5—H5A	0.9800	C21—C22	1.3911 (12)
C5—H5B	0.9800	C21—H21	0.9500
C5—H5C	0.9800	C22—C23	1.3964 (11)
B1—C18	1.6388 (12)	C22—H22A	0.9500
B1—C24	1.6423 (12)	C23—H23A	0.9500
B1—C12	1.6436 (12)	C24—C29	1.4051 (12)
B1—C6	1.6458 (12)	C24—C25	1.4085 (11)
C6—C11	1.3988 (11)	C25—C26	1.3916 (12)
C6—C7	1.4114 (11)	C25—H25A	0.9500
C7—C8	1.3926 (12)	C26—C27	1.3927 (14)
C7—H7A	0.9500	C26—H26A	0.9500
C8—C9	1.3891 (13)	C27—C28	1.3886 (14)
C8—H8A	0.9500	C27—H27A	0.9500
C9—C10	1.3885 (13)	C28—C29	1.3999 (12)
C9—H9A	0.9500	C28—H28A	0.9500
C10—C11	1.3996 (12)	C29—H29A	0.9500
C10—H10A	0.9500		
			
C1—N1—C3	120.30 (8)	C6—C11—C10	122.44 (8)
C1—N1—C2	121.86 (8)	C6—C11—H11A	118.8
C3—N1—C2	116.21 (8)	C10—C11—H11A	118.8
C1—N2—C4	121.58 (8)	C17—C12—C13	115.15 (7)
C1—N2—C5	121.62 (9)	C17—C12—B1	124.75 (7)
C4—N2—C5	115.04 (8)	C13—C12—B1	119.76 (7)
C1—N3—H30	119.6 (10)	C14—C13—C12	122.98 (8)
C1—N3—H31	120.5 (10)	C14—C13—H13A	118.5
H30—N3—H31	119.9 (14)	C12—C13—H13A	118.5
N2—C1—N1	120.69 (8)	C15—C14—C13	120.08 (8)
N2—C1—N3	119.81 (9)	C15—C14—H14A	120.0
N1—C1—N3	119.51 (8)	C13—C14—H14A	120.0
N1—C2—H2A	109.5	C16—C15—C14	118.80 (8)
N1—C2—H2B	109.5	C16—C15—H15A	120.6
H2A—C2—H2B	109.5	C14—C15—H15A	120.6
N1—C2—H2C	109.5	C15—C16—C17	120.34 (8)
H2A—C2—H2C	109.5	C15—C16—H16A	119.8
H2B—C2—H2C	109.5	C17—C16—H16A	119.8
N1—C3—H3A	109.5	C16—C17—C12	122.61 (8)
N1—C3—H3B	109.5	C16—C17—H17A	118.7
H3A—C3—H3B	109.5	C12—C17—H17A	118.7
N1—C3—H3C	109.5	C23—C18—C19	115.29 (7)
H3A—C3—H3C	109.5	C23—C18—B1	123.60 (7)
H3B—C3—H3C	109.5	C19—C18—B1	120.89 (7)
N2—C4—H4A	109.5	C20—C19—C18	122.86 (8)
N2—C4—H4B	109.5	C20—C19—H19	118.6
H4A—C4—H4B	109.5	C18—C19—H19	118.6
N2—C4—H4C	109.5	C21—C20—C19	120.08 (8)
H4A—C4—H4C	109.5	C21—C20—H20A	120.0
H4B—C4—H4C	109.5	C19—C20—H20A	120.0
N2—C5—H5A	109.5	C22—C21—C20	118.81 (8)
N2—C5—H5B	109.5	C22—C21—H21	120.6
H5A—C5—H5B	109.5	C20—C21—H21	120.6
N2—C5—H5C	109.5	C21—C22—C23	120.25 (8)
H5A—C5—H5C	109.5	C21—C22—H22A	119.9
H5B—C5—H5C	109.5	C23—C22—H22A	119.9
C18—B1—C24	111.55 (6)	C22—C23—C18	122.68 (8)
C18—B1—C12	112.71 (6)	C22—C23—H23A	118.7
C24—B1—C12	103.43 (6)	C18—C23—H23A	118.7
C18—B1—C6	105.47 (6)	C29—C24—C25	115.45 (8)
C24—B1—C6	110.52 (6)	C29—C24—B1	123.91 (7)
C12—B1—C6	113.31 (6)	C25—C24—B1	120.40 (7)
C11—C6—C7	115.29 (7)	C26—C25—C24	122.75 (8)
C11—C6—B1	125.30 (7)	C26—C25—H25A	118.6
C7—C6—B1	119.40 (7)	C24—C25—H25A	118.6
C8—C7—C6	123.03 (8)	C25—C26—C27	120.06 (9)
C8—C7—H7A	118.5	C25—C26—H26A	120.0
C6—C7—H7A	118.5	C27—C26—H26A	120.0
C9—C8—C7	119.84 (8)	C28—C27—C26	119.13 (8)
C9—C8—H8A	120.1	C28—C27—H27A	120.4
C7—C8—H8A	120.1	C26—C27—H27A	120.4
C10—C9—C8	118.95 (8)	C27—C28—C29	120.02 (9)
C10—C9—H9A	120.5	C27—C28—H28A	120.0
C8—C9—H9A	120.5	C29—C28—H28A	120.0
C9—C10—C11	120.43 (8)	C28—C29—C24	122.58 (8)
C9—C10—H10A	119.8	C28—C29—H29A	118.7
C11—C10—H10A	119.8	C24—C29—H29A	118.7
			
C4—N2—C1—N1	−162.47 (9)	C14—C15—C16—C17	−1.56 (14)
C5—N2—C1—N1	33.40 (13)	C15—C16—C17—C12	0.81 (14)
C4—N2—C1—N3	17.62 (13)	C13—C12—C17—C16	0.93 (12)
C5—N2—C1—N3	−146.51 (9)	B1—C12—C17—C16	174.07 (8)
C3—N1—C1—N2	−160.77 (9)	C24—B1—C18—C23	−18.25 (10)
C2—N1—C1—N2	34.35 (13)	C12—B1—C18—C23	−134.10 (8)
C3—N1—C1—N3	19.15 (13)	C6—B1—C18—C23	101.77 (8)
C2—N1—C1—N3	−145.74 (9)	C24—B1—C18—C19	167.38 (7)
C18—B1—C6—C11	120.33 (9)	C12—B1—C18—C19	51.53 (10)
C24—B1—C6—C11	−118.97 (9)	C6—B1—C18—C19	−72.59 (9)
C12—B1—C6—C11	−3.41 (11)	C23—C18—C19—C20	1.29 (12)
C18—B1—C6—C7	−58.20 (9)	B1—C18—C19—C20	176.11 (7)
C24—B1—C6—C7	62.51 (9)	C18—C19—C20—C21	−0.06 (13)
C12—B1—C6—C7	178.06 (7)	C19—C20—C21—C22	−1.08 (12)
C11—C6—C7—C8	1.24 (13)	C20—C21—C22—C23	0.91 (12)
B1—C6—C7—C8	179.91 (8)	C21—C22—C23—C18	0.40 (12)
C6—C7—C8—C9	0.29 (14)	C19—C18—C23—C22	−1.47 (12)
C7—C8—C9—C10	−1.19 (14)	B1—C18—C23—C22	−176.12 (7)
C8—C9—C10—C11	0.50 (15)	C18—B1—C24—C29	133.43 (8)
C7—C6—C11—C10	−1.94 (13)	C12—B1—C24—C29	−105.16 (8)
B1—C6—C11—C10	179.48 (9)	C6—B1—C24—C29	16.43 (11)
C9—C10—C11—C6	1.14 (15)	C18—B1—C24—C25	−52.52 (10)
C18—B1—C12—C17	13.50 (11)	C12—B1—C24—C25	68.88 (9)
C24—B1—C12—C17	−107.12 (9)	C6—B1—C24—C25	−169.53 (7)
C6—B1—C12—C17	133.19 (8)	C29—C24—C25—C26	−1.38 (12)
C18—B1—C12—C13	−173.65 (7)	B1—C24—C25—C26	−175.91 (8)
C24—B1—C12—C13	65.73 (9)	C24—C25—C26—C27	0.66 (13)
C6—B1—C12—C13	−53.96 (10)	C25—C26—C27—C28	0.67 (13)
C17—C12—C13—C14	−1.98 (12)	C26—C27—C28—C29	−1.20 (14)
B1—C12—C13—C14	−175.49 (8)	C27—C28—C29—C24	0.42 (14)
C12—C13—C14—C15	1.30 (14)	C25—C24—C29—C28	0.84 (12)
C13—C14—C15—C16	0.55 (14)	B1—C24—C29—C28	175.15 (8)
